# Supplementary material for: Resolving the full spectrum of human genome variation using Linked-Reads
Source: Genome Res. 2019 Apr;29(4):635–45. doi: 10.1101/gr.234443.118 (PMC6442396; doi:10.1101/gr.234443.118)
Supplement: Supplemental Material [file supp_gr.234443.118_Supplemental_longranger-2.2.zip › longranger-2.2.tar.bz2!/lr2.2-public/lib/stan/cmdstan/stan/doc/api/html/multi__gp_8hpp_source.html]

Stan: src/stan/prob/distributions/multivariate/continuous/multi\_gp.hpp Source File


|  |  |
| --- | --- |
|  | Stan  2.5.0  probability, sampling & optimization |


- Main Page
- Related Pages
- Namespaces
- Classes
- Files

- File List
- File Members

All Classes Namespaces Files Functions Variables Typedefs Enumerations Enumerator Friends Macros Pages

- src
- stan
- prob
- distributions
- multivariate
- continuous

multi\_gp.hpp

Go to the documentation of this file.

1 #ifndef STAN\_\_PROB\_\_DISTRIBUTIONS\_\_MULTIVARIATE\_\_CONTINUOUS\_\_MULTI\_GP\_HPP

2 #define STAN\_\_PROB\_\_DISTRIBUTIONS\_\_MULTIVARIATE\_\_CONTINUOUS\_\_MULTI\_GP\_HPP

3

4 #include <stan/math/matrix\_error\_handling.hpp>

5 #include <stan/math/error\_handling.hpp>

6 #include <stan/math/error\_handling/dom\_err.hpp>

7 #include <stan/prob/constants.hpp>

8 #include <stan/prob/traits.hpp>

9 #include <stan/agrad/rev.hpp>

10 #include <stan/meta/traits.hpp>

11 #include <stan/agrad/rev/matrix.hpp>

12 #include <stan/math/matrix/log.hpp>

13 #include <stan/math/matrix/multiply.hpp>

14 #include <stan/math/matrix/sum.hpp>

15

16 #include <stan/math/matrix/log\_determinant\_ldlt.hpp>

17 #include <stan/math/matrix/trace\_gen\_inv\_quad\_form\_ldlt.hpp>

18 #include <stan/math/error\_handling/matrix/check\_ldlt\_factor.hpp>

19

20 namespace stan {

21  namespace prob {

22  // MultiGP(y|Sigma,w) [y.rows() = w.size(), y.cols() = Sigma.rows();

23  // Sigma symmetric, non-negative, definite]

42  template <bool propto,

43  typename T\_y, typename T\_covar, typename T\_w>

44  typename boost::math::tools::promote\_args<T\_y,T\_covar,T\_w>::type

45  multi\_gp\_log(const Eigen::Matrix<T\_y,Eigen::Dynamic,Eigen::Dynamic>& y,

46  const Eigen::Matrix<T\_covar,Eigen::Dynamic,Eigen::Dynamic>& Sigma,

47  const Eigen::Matrix<T\_w,Eigen::Dynamic,1>& w) {

48  static const char\* function = "stan::prob::multi\_gp\_log(%1%)";

49  typedef typename boost::math::tools::promote\_args<T\_y,T\_covar,T\_w>::type T\_lp;

50  T\_lp lp(0.0);

51

52  using stan::math::sum;

53  using stan::math::log;

54  using stan::math::LDLT\_factor;

55  using stan::math::log\_determinant\_ldlt;

56  using stan::math::trace\_gen\_inv\_quad\_form\_ldlt;

57

58  using stan::math::check\_size\_match;

59  using stan::math::check\_positive\_finite;

60  using stan::math::check\_positive;

61  using stan::math::check\_finite;

62  using stan::math::check\_symmetric;

63  using stan::math::check\_ldlt\_factor;

64  using stan::math::check\_not\_nan;

65

66  check\_size\_match(function,

67  Sigma.rows(), "Rows of kernel matrix",

68  Sigma.cols(), "columns of kernel matrix",

69  &lp);

70  check\_positive(function, Sigma.rows(), "Kernel rows", &lp);

71  check\_finite(function, Sigma, "Kernel", &lp);

72  check\_symmetric(function, Sigma, "Kernel", &lp);

73

74  LDLT\_factor<T\_covar,Eigen::Dynamic,Eigen::Dynamic> ldlt\_Sigma(Sigma);

75  check\_ldlt\_factor(function,ldlt\_Sigma,"LDLT\_Factor of Sigma",&lp);

76

77  check\_size\_match(function,

78  y.rows(), "Size of random variable (rows y)",

79  w.size(), "Size of kernel scales (w)",

80  &lp);

81  check\_size\_match(function,

82  y.cols(), "Size of random variable",

83  Sigma.rows(), "rows of covariance parameter",

84  &lp);

85  check\_positive\_finite(function, w, "Kernel scales", &lp);

86  check\_finite(function, y, "Random variable", &lp);

87

88  if (y.rows() == 0)

89  return lp;

90

91  if (include\_summand<propto>::value) {

92  lp += NEG\_LOG\_SQRT\_TWO\_PI \* y.rows() \* y.cols();

93  }

94

95  if (include\_summand<propto,T\_covar>::value) {

96  lp -= 0.5 \* log\_determinant\_ldlt(ldlt\_Sigma) \* y.rows();

97  }

98

99  if (include\_summand<propto,T\_w>::value) {

100  lp += (0.5 \* y.cols()) \* sum(log(w));

101  }

102

103  if (include\_summand<propto,T\_y,T\_w,T\_covar>::value) {

104  Eigen::Matrix<T\_w,Eigen::Dynamic,Eigen::Dynamic> w\_mat(w.asDiagonal());

105  Eigen::Matrix<T\_y,Eigen::Dynamic,Eigen::Dynamic> yT(y.transpose());

106  lp -= 0.5 \* trace\_gen\_inv\_quad\_form\_ldlt(w\_mat,ldlt\_Sigma,yT);

107  }

108

109  return lp;

110  }

111

112  template <typename T\_y, typename T\_covar, typename T\_w>

113  inline

114  typename boost::math::tools::promote\_args<T\_y,T\_covar,T\_w>::type

115  multi\_gp\_log(const Eigen::Matrix<T\_y,Eigen::Dynamic,Eigen::Dynamic>& y,

116  const Eigen::Matrix<T\_covar,Eigen::Dynamic,Eigen::Dynamic>& Sigma,

117  const Eigen::Matrix<T\_w,Eigen::Dynamic,1>& w) {

118  return multi\_gp\_log<false>(y,Sigma,w);

119  }

120  }

121 }

122

123 #endif

matrix.hpp

dom\_err.hpp

stan::prob::multi\_gp\_log

boost::math::tools::promote\_args< T\_y, T\_covar, T\_w >::type multi\_gp\_log(const Eigen::Matrix< T\_y, Eigen::Dynamic, Eigen::Dynamic > &y, const Eigen::Matrix< T\_covar, Eigen::Dynamic, Eigen::Dynamic > &Sigma, const Eigen::Matrix< T\_w, Eigen::Dynamic, 1 > &w)

The log of a multivariate Gaussian Process for the given y, Sigma, and w.

**Definition:** multi\_gp.hpp:45

traits.hpp

stan::math::check\_positive\_finite

bool check\_positive\_finite(const char \*function, const T\_y &y, const char \*name, T\_result \*result)

**Definition:** check\_positive\_finite.hpp:12

stan::math::check\_size\_match

bool check\_size\_match(const char \*function, T\_size1 i, const char \*name\_i, T\_size2 j, const char \*name\_j, T\_result \*result)

**Definition:** check\_size\_match.hpp:13

error\_handling.hpp

stan::math::check\_finite

bool check\_finite(const char \*function, const T\_y &y, const char \*name, T\_result \*result)

Checks if the variable y is finite.

**Definition:** check\_finite.hpp:52

log.hpp

matrix\_error\_handling.hpp

constants.hpp

stan::agrad::sum

fvar< T > sum(const Eigen::Matrix< fvar< T >, R, C > &m)

**Definition:** sum.hpp:14

traits.hpp

stan::math::check\_symmetric

bool check\_symmetric(const char \*function, const Eigen::Matrix< T\_y, Eigen::Dynamic, Eigen::Dynamic > &y, const char \*name, T\_result \*result)

Return true if the specified matrix is symmetric.

**Definition:** check\_symmetric.hpp:31

stan::math::check\_ldlt\_factor

bool check\_ldlt\_factor(const char \*function, stan::math::LDLT\_factor< T, R, C > &A, const char \*name, T\_result \*result)

Return true if the underlying matrix is positive definite.

**Definition:** check\_ldlt\_factor.hpp:24

check\_ldlt\_factor.hpp

stan::math::trace\_gen\_inv\_quad\_form\_ldlt

boost::enable\_if\_c<!stan::is\_var< T1 >::value &&!stan::is\_var< T2 >::value &&!stan::is\_var< T3 >::value, typename boost::math::tools::promote\_args< T1, T2, T3 >::type >::type trace\_gen\_inv\_quad\_form\_ldlt(const Eigen::Matrix< T1, R1, C1 > &D, const stan::math::LDLT\_factor< T2, R2, C2 > &A, const Eigen::Matrix< T3, R3, C3 > &B)

**Definition:** trace\_gen\_inv\_quad\_form\_ldlt.hpp:28

stan::prob::include\_summand

Template metaprogram to calculate whether a summand needs to be included in a proportional (log) prob...

**Definition:** traits.hpp:35

rev.hpp

stan::math::LDLT\_factor

**Definition:** LDLT\_factor.hpp:16

log\_determinant\_ldlt.hpp

trace\_gen\_inv\_quad\_form\_ldlt.hpp

stan::math::check\_positive

bool check\_positive(const char \*function, const T\_y &y, const char \*name, T\_result \*result)

**Definition:** check\_positive.hpp:54

stan::math::log

Eigen::Matrix< T, Rows, Cols > log(const Eigen::Matrix< T, Rows, Cols > &m)

Return the element-wise logarithm of the matrix or vector.

**Definition:** log.hpp:16

stan::math::sum

double sum(std::vector< double > &x)

**Definition:** sum.hpp:10

stan::math::check\_not\_nan

bool check\_not\_nan(const char \*function, const T\_y &y, const char \*name, T\_result \*result)

Checks if the variable y is nan.

**Definition:** check\_not\_nan.hpp:56

stan::agrad::log\_determinant\_ldlt

var log\_determinant\_ldlt(stan::math::LDLT\_factor< var, R, C > &A)

**Definition:** log\_determinant\_ldlt.hpp:48

stan::math::log\_determinant\_ldlt

T log\_determinant\_ldlt(stan::math::LDLT\_factor< T, R, C > &A)

**Definition:** log\_determinant\_ldlt.hpp:12

stan::agrad::trace\_gen\_inv\_quad\_form\_ldlt

boost::enable\_if\_c< boost::is\_same< T1, var >::value||boost::is\_same< T2, var >::value||boost::is\_same< T3, var >::value, var >::type trace\_gen\_inv\_quad\_form\_ldlt(const Eigen::Matrix< T1, R1, C1 > &D, const stan::math::LDLT\_factor< T2, R2, C2 > &A, const Eigen::Matrix< T3, R3, C3 > &B)

Compute the trace of an inverse quadratic form.

**Definition:** trace\_gen\_inv\_quad\_form\_ldlt.hpp:25

sum.hpp

stan::agrad::log

fvar< T > log(const fvar< T > &x)

**Definition:** log.hpp:15

multiply.hpp


---

[ Stan Home Page ]

*© 2011–2014,
Stan Development Team.*
